# Saunders’ Medical Hand-Atlases

**Published:** 1902-04

**Authors:** 


SAUNDERS' MEDICAL HAND-ATLASES.—Atlas and Epitome of Special Pathologic Histology. By Docent Dr. Hermann Dürck, of the Pathologic Institute of Munich. Edited by Ludvig Kektoen, M. D., Professor of Pathology in Rush Medical College, Chicago. Vol. II.—Liver; Urinary Organs; Sexual Organs; Nervous System; Skin; Muscles; Bones. With 123 colored illustrations on 60 lithographic plates and 192 pages of text. Philadelphia and London: W. B. Saunders & Co. 1901. Cloth, $3.00 net.

Too much cannot be said in praise of the superb lithographic plates and other colored illustrations contained in this work. As is true of most of the other Saunders hand-atlases, the illustrations are really worth many times the price charged for the entire work. Unfortunately, so much cannot be said of the text of the particular volume. It is entirely too condensed to be of much real value for either study or reference. What there is of it, however, is fairly good. R.

